# Cow Placenta Extract Ameliorates Cyclophosphamide-Induced Intestinal Damage by Enhancing the Intestinal Barrier, Improving Immune Function, and Restoring Intestinal Microbiota

**DOI:** 10.3390/vetsci11100505

**Published:** 2024-10-14

**Authors:** Yuquan Zhao, Zeru Zhang, Anguo Tang, Zhi Zeng, Weijian Zheng, Yuxin Luo, Yixin Huang, Xinyi Dai, Wei Lu, Lei Fan, Liuhong Shen

**Affiliations:** 1 The Key Laboratory of Animal Disease and Human Health of Sichuan Province, The Medical Research Center for Cow Disease, College of Veterinary Medicine, Sichuan Agricultural University, Chengdu 611130, China; zhaoyuquan@stu.sicau.edu.cn (Y.Z.); zhangzr@stu.sicau.edu.cn (Z.Z.); tangag@haid.com.cn (A.T.); 2023303092@stu.sicau.edu.cn (Z.Z.); zhengweijian@stu.sicau.edu.cn (W.Z.); llyuxx@stu.sicau.edu.cn (Y.L.); yxhuang@sicau.edu.cn (Y.H.); 2 Party School of the Communist Party of China Yaan Municipal Committee, Yaan 625014, China; xinyidai.yaswdx@outlook.com; 3 Jiangsu Agri-Animal Husbandry Vocational College, Taizhou 225300, China; 2002010183@jsahvc.edu.cn; 4 Department of Pharmacology, Wuhan University School of Basic Medical Sciences, Wuhan 430071, China

**Keywords:** immunosuppression, cow placenta extract, cyclophosphamide, intestinal health, gut microbiota

## Abstract

**Simple Summary:**

The intestinal tract is one of the most important barriers in an organism. Cyclophosphamide can lead to the destruction of the structural integrity of the intestinal barrier, causing immunosuppression, oxidative stress, and microbiota disorders. Conversely, cow placenta extract can improve the immunity and antioxidant power of the organism, etc. Therefore, it is important to investigate the protective effects of cow placenta extract on the intestinal barrier, intestinal immune function, and intestinal microbiota caused by cyclophosphamide to provide a new approach for its clinical application in the treatment of intestinal injury caused by immunosuppression.

**Abstract:**

Immunosuppression undermines intestinal barrier integrity. Cow placenta extract (CPE) primarily consists of active peptides with immunomodulatory and antioxidant effects. This study aimed to examine the preventive effect of CPE against intestinal damage induced by cyclophosphamide (Cy) in immunosuppressed mice. Thirty-six mice were randomly allocated into three groups: control group (C), model group (M), and treatment group (CPE). The mice in the CPE group were provided with 1500 mg/kg/day of CPE via gavage. In the last 3 days, mice in the groups M and CPE received intraperitoneal injections of 80 mg/kg/day of Cy. The results showed that CPE improved intestinal barrier function by decreasing serum d-Lactate (D-LA) levels and diamine oxidase (DAO) activity, while elevating the relative expression of *Occludin*, *zonula occludens-1* (*ZO-1*), and *mucin-2* (*MUC-2*) mRNA. Additionally, CPE improved the immune organ index and elevated the levels of secretory immunoglobulin A (sIgA), superoxide dismutase (SOD), interleukin-1beta (IL-1β), interleukin-4 (IL-4), interleukin-10 (IL-10), and tumor necrosis factor-α (TNF-α) in the intestine, thereby enhancing intestinal mucosal immune function. Furthermore, CPE improved the diversity of intestinal microbiota and increased the abundance of *Candidatus_Saccharimonas*, *Psychrobacter*, and *Enterorhabdus*, which promoted the proper functioning of the intestines. These findings suggest that CPE effectively ameliorates Cy-induced intestinal damage by enhancing the intestinal barrier, improving immune function, and restoring intestinal microbiota.

## 1. Introduction

The intestinal tract, known as the primary digesting organ and the largest immune organ, acts as the body’s primary barrier against the invasion of pathogens due to its extensive surface area and abundance of immune cells [1]. Nevertheless, the integrity of the intestinal barrier can be undermined by various causes, such as immunosuppression, oxidative stress, and inflammation [2]. Immunosuppression, in particular, compromises the integrity of the intestinal barrier, thereby enabling the transfer of pathogenic microorganisms and other deleterious substances into the intestine, which in turn leads to intestinal inflammation and microbial imbalance [3]. Cyclophosphamide (Cy) is frequently used to construct immunosuppression models. It suppresses cellular and humoral immunity by altering DNA structure, diminishing the quantity of lymphocytes and macrophages, and decreasing the weight of immune organs, leading to immunosuppression [4]. Prolonged or high-dose administration of Cy (80 or 100 mg/kg) has been shown to impair the integrity of the intestinal mucosa, disrupt the morphology of the intestinal villi, increase the permeability of the intestinal mucosal barrier [5], induce oxidative damage within intestinal tissues [6], and lead to immune dysfunction [7]. In addition, Cy can also change the intestinal microbes and increase the number of potential pathogenic bacteria [8]. In summary, Cy induces significant immunosuppression and impairs the normal functioning of the intestine, which is essential for animal production. Thus, it is crucial to discover appropriate pharmaceuticals to alleviate the harm inflicted by immunosuppression on the intestines.

Bioactive peptides demonstrate a broad spectrum of biological activities that beneficially impact intestinal homeostasis by modulating barrier function, immune response, and the composition of the intestinal microbiota [9,10,11]. The placenta, often referred to as “Ziheche” in traditional Chinese medicine, is predominantly composed of bioactive peptides that have effects on promoting cell proliferation, immunoregulation, and antioxidants [12]. Studies have shown that porcine placenta extract can boost the immune system in mice by boosting the immune organ index and levels of immune-related cytokines [13]. Immunomodulatory peptides isolated from bovine placenta exhibit a dose-dependent stimulatory effect on lymphocyte proliferation [14]. The cow placenta is an abundant natural resource that has not been efficiently used. Thus far, we have established the optimal extraction conditions and identified the main components of cow placenta extract by mass spectrometry, which has proven to possess good antioxidative and immunomodulatory effects [15,16]. This is closely connected to the requirement for therapy of Cy-induced immunosuppression and intestinal injury. This work seeks to examine the protective effect of cow placenta extract (CPE) on the intestinal barrier, immunology, and microbiota in immunosuppressed mice induced by Cy. This offers a promising approach to mitigate the intestinal damage caused by immunosuppression.

## 2. Materials and Methods

### 2.1. Preparation of CPE

CPE was prepared according to the previous research methods of our group [15]. Cow placenta was hydrolyzed with papain, after which the supernatant was collected and freeze-dried. The components of CPE were identified by mass spectrometry technology to contain 128 peptides, with peptide lengths ranging from 7 to 25 amino acids and molecular weights in the range of 800 to 3100 Da [16].

### 2.2. Animals and Experimental Design

Thirty-six male Kunming mice, aged 8 weeks and weighing 43.75 ± 0.19 g, were acquired from Beijing Sibeifu Biotechnology Co., Ltd. (Beijing, China). Following one week of adaptive feeding, the mice were randomly allocated into three groups (*n* = 12): a control group (C), a Cy model group (M), and the cow placental extract treatment group (CPE). The mice in the CPE group received 1500 mg/kg/day of CPE via gavage. Concurrently, groups C and M received an equivalent volume of physiological saline orally. The experimental period was 28 days, and in the last 3 days, groups M and CPE were administered 80 mg/kg/day [17] of Cy intraperitoneally, while group C received an equivalent volume of saline injection. The selection of the CPE dosage was based on preliminary experiments conducted across four concentration gradients (500, 1000, 1500, and 2000 mg/kg). The results of the preliminary experiments are included in the Supplementary Materials.

The criteria for successful model establishment included the following: the mice exhibited decreased body weight and immune organ index, structural disruption of intestinal villi with variable lengths and sparse arrangement, reduced levels of intestinal immune-related cytokines, and an impaired intestinal barrier characterized by increased permeability. The above results were consistent with other reports [18,19,20].

### 2.3. Preparation of Serum and Tissue

The mice underwent a 12 h fasting period following the final treatment and were anesthetized via inhalation of 2% isoflurane. Blood had been taken from the mouse’s eyes, permitted to stand for 30 min, and centrifuged at 4 °C and 9200× *g* for 5 min, after which the serum was isolated and preserved. The intestines were collected for subsequent tests by laparotomy. The spleen and thymus were collected and weighed, and the immune organ index was computed using the subsequent formula:

$$Immune\;organ\;index\;(mg/g)=\frac{Spleen/Thymus\;weight\;(mg)}{Body\;weight\;(g)}$$

### 2.4. Measurement of Serum Cytokines Level

The levels of diamine oxidase (DAO) and d-Lactate (D-LA) in serum samples were measured according to the instructions provided with the assay kit (Shanghai Enzyme-linked, Shanghai, China).

### 2.5. Histomorphometry of Small Intestine

The tissues of the duodenum, jejunum, and ileum were fixed in 10% formaldehyde and later imbedded in paraffin blocks. These blocks were sliced and dyed with hematoxylin and eosin. Images were acquired using an optical microscope at a magnification of 100×. The lengths of the intestinal villi and the depths of the crypts were quantified using ImagePro Plus 6.0 software.

### 2.6. Determination of Cytokines, sIgA and β-DF in Jejunum Tissue

The jejunal tissue was weighed and homogenized thoroughly in a phosphate-buffered saline (PBS) glass homogenizer under ice bath conditions at a mass-to-volume ratio of 1:9. After homogenization, the supernatant was obtained using centrifugation at 4600× *g* for 20 min at 4 °C. The levels of superoxide dismutase (SOD), malondialdehyde (MDA), tumor necrosis factor-α (TNF-α), interleukin-1beta (IL-1β), interleukin-4 (IL-4), interleukin-10 (IL-10), secretory immunoglobulin A (sIgA), and β-DF (β-Defensins) were determined according to the instructions provided with the assay kit (Shanghai Enzyme-linked, Shanghai, China).

### 2.7. Determination of Intestine mRNA

Total RNA was isolated from jejunal tissue utilizing the TRIzol RNA extraction reagent (Servicebio, Wuhan, China). Subsequently, cDNA was synthesized using the FastQuant RT Kit (Servicebio, Wuhan, China) and amplified via PCR on a Bio-Rad instrument (Hercules, CA, USA). GAPDH functioned as the internal reference gene for real-time quantitative PCR analysis. The data were processed using the 2^−ΔΔCT^ method. Primers were synthesized by Shanghai Sangon Biotech (Shanghai) Co., Ltd. (Shanghai, China). The sequences are detailed in Table 1.

### 2.8. Microbiota 16S rDNA Amplicon Sequencing

The whole DNA of the fecal microbiome was isolated using the CTAB method. Subsequently, PCR amplification of the microbial 16S rDNA gene’s V3–V4 region was conducted, employing the forward primer 341F (5′-CCTACGGGNGGCWGCAG-3′) and the reverse primer 805R (5′-GACTACHVGGGTATCTAATCC-3′). The quality of the resulting library was assessed using the Agilent 2100 Bioanalyzer and the Illumina library quantification kit. Paired-end sequencing (2 × 250 bp) was then performed on a NovaSeq 6000 sequencer at Lianchuan Biotech Co., Ltd. (Hangzhou, China). For microbial community analysis, the Kruskal–Wallis rank sum test was employed to identify species with significant differences between groups. Subsequently, the Wilcoxon rank sum test was conducted to determine whether all subspecies exhibiting significant differences converged at the same taxonomic level. Additionally, linear discriminant analysis (LDA) was used, along with the LEfSe algorithm, to screen for target differential species based on an LDA score ≥ 3 and *p* < 0.05.

### 2.9. Statistical Analysis

All data were expressed as mean ± standard deviation (SD). One-way analysis of variance and Duncan’s multiple range test were employed to assess the significance of differences between groups. Statistical analysis was conducted using SPSS statistical software version 23 (SPSS Inc., Chicago, IL, USA). A *p*-value of less than 0.05 was deemed statistically significant.

## 3. Results

### 3.1. Results of Body Weight and Immune Organ Index of Mice in Each Group

**Figure 1:**
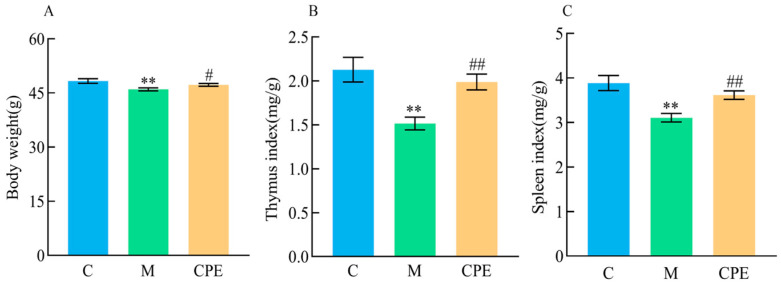
Test results of body weight and immune organ index in experimental mice. (**A**) Mouse body weight on the 28th day; (**B**) thymic index in each group; (**C**) spleen index in each group. ** indicates that group M exhibited statistical significance compared to group C. ** indicates *p* < 0.01; # indicates that group CPE exhibited statistical significance compared to group M. # indicates *p* < 0.05, ## indicates *p* < 0.01 (*n* = 12).

The body weight of mice increased with the duration of feeding throughout the 21 days preceding the trial, with no significant differences (*p* > 0.05) noted between groups (Table 2). On day 28, the body weight of group M was significantly lower (*p* < 0.01) than that of group C, whereas the body weight of group CPE was significantly greater (*p* < 0.05) than that of group M (Figure 1A). The thymus and spleen index in group M was significantly lower (*p* < 0.01) than that in group C, whereas the index in group CPE was significantly greater (*p* < 0.01) than that in group M (Figure 1B,C).

### 3.2. Detection Results of Intestinal Permeability Index in Each Group of Experimental Mice

**Figure 2:**
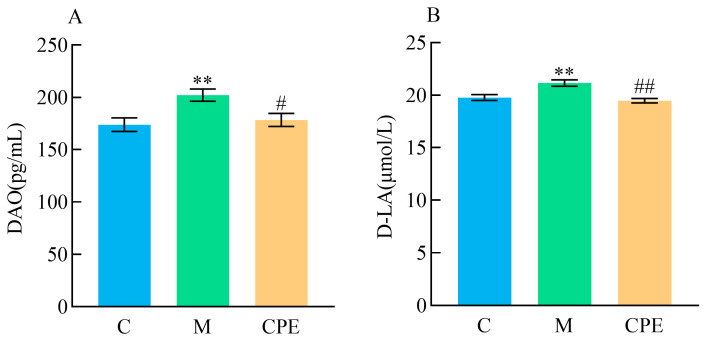
Detection results of intestinal permeability index in each group of experimental mice. (**A**) Diamine oxidase detection results in each group. (**B**) D-lactate results in each group. ** indicates that group M exhibited statistical significance compared to group C. ** indicates *p* < 0.01; # indicates that group CPE exhibited statistical significance compared to group M. # indicates *p* < 0.05, ## indicates *p* < 0.01 (*n* = 12).

Figure 2 illustrates the concentrations of DAO and D-LA among various groups of mice. In comparison to group C, DAO and D-LA levels exhibited a highly significant rise (*p* < 0.01) in group M. In comparison to group M, serum DAO levels in group CPE were dramatically reduced (*p* > 0.05), while D-LA levels were markedly diminished (*p* < 0.01).

### 3.3. Results of Intestinal Sections of Mice in Each Group

**Figure 3:**
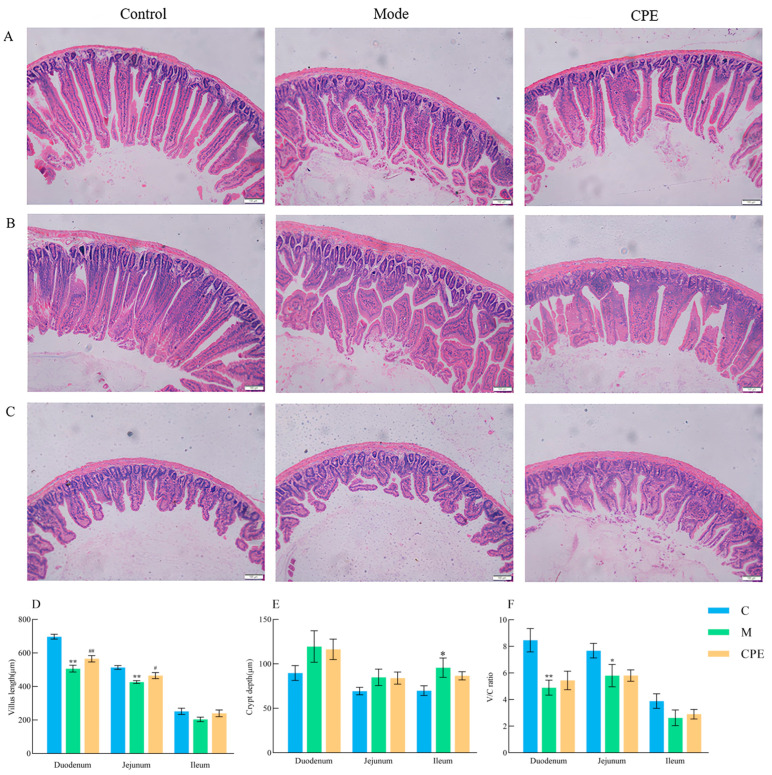
Results of intestinal sections of mice in each group. (**A**) The effect of CPE on the morphological characteristics of the duodenum in each group. (**B**) The influence of CPE on the morphological characteristics of the jejunum in each group. (**C**) The influence of CPE on the morphological characteristics of the ileum in each group; scale: 100 µm, magnification: 100×. (**D**) The effect of CPE on the height of small intestinal villi in each group. (**E**) Depth of small intestinal villous crypt in each group. (**F**) The ratio of small intestinal villus height to crypt depth in each group. * indicates that group M exhibited statistical significance compared to group C. * indicates *p* < 0.05, ** indicates *p* < 0.01. # indicates that group CPE exhibited statistical significance compared to group M. # indicates *p* < 0.05, ## indicates *p* < 0.01 (*n* = 10).

H&E staining was employed to evaluate intestine morphological and structural damage in mice (Figure 3). The intestinal mucosal tissue in the control group appeared structurally intact, with regularly distributed villi displaying distinct boundaries. Conversely, in group M, the intestinal mucosal tissue displayed damage, characterized by villi of inconsistent lengths and sparse configurations. Nonetheless, the administration of CPE enhanced the structure and morphology of the small intestine mucosal tissue to differing extents. Furthermore, the heights of duodenal and jejunal villi, along with the villus height/crypt depth ratios (V/C ratios), were significantly diminished (*p* < 0.01 or *p* < 0.05) in group M relative to group C, although the depth of the cecum crypt was dramatically increased (*p* < 0.05). Moreover, the height of duodenal and jejunal villi was significantly increased (*p* < 0.01 or *p* < 0.05) in group CPE relative to group M.

### 3.4. Detection Results of sIgA, β-DF, Oxidation, and Cytokines in Intestinal Tissue of Mice in Each Group

**Figure 4:**
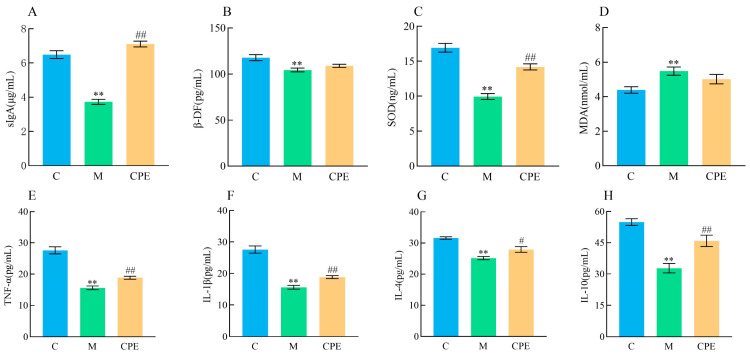
Detection of sIgA, β-DF, and cytokines in intestinal tissue of mice in each group. (**A**) Detection results of sIgA in each group. (**B**) Detection results of β-DF in each group. (**C**) Detection results of SOD in each group. (**D**) Detection results of MDA in each group. (**E**) Detection results of TNF-α in each group. (**F**) Detection results of IL-1β in each group. (**G**) Detection results of IL-4 in each group. (**H**) Detection results of IL-10 in each group. ** indicates that group M exhibited statistical significance compared to group C. ** indicates *p* < 0.01. # indicates that group CPE exhibited statistical significance compared to group M. # indicates *p* < 0.05, ## indicates *p* < 0.01 (*n* = 12).

As illustrated in Figure 4, the activity of SOD and the levels of sIgA, β-DF, TNF-α, IL-1β, IL-4, and IL-10 were markedly diminished (*p* < 0.01), while the level of MDA was dramatically elevated (*p* < 0.01) in group M compared with group C. The administration of CPE significantly enhanced (*p* < 0.01 or *p* < 0.05) the activity of SOD and the levels of sIgA, β-DF, TNF-α, IL-1β, IL-4, and IL-10. Despite a declining trend in MDA levels, no significant difference was observed (*p* > 0.05).

### 3.5. Detection Results of mRNA Relative Expression Levels of Intestinal Barrier Related Genes in Mice from Various Experimental Groups

**Figure 5:**
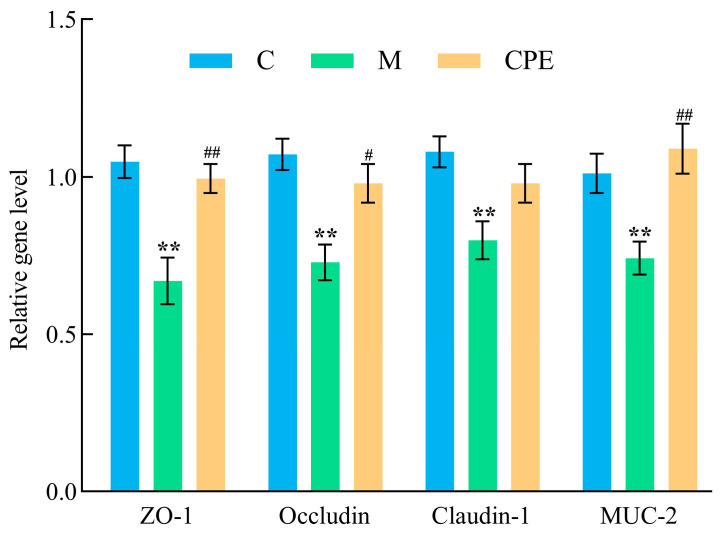
Detection results of relative expression levels of intestinal barrier genes and mucin 2 (*MUC2*) mRNA in mice from various experimental groups. ** indicates that group M exhibited statistical significance compared to group C. ** indicates *p* < 0.01. # indicates that group CPE exhibited statistical significance compared to group M. # indicates *p* < 0.05, ## indicates *p* < 0.01 (*n* = 6).

To further assess the extent of damage to the intestinal barrier, we analyzed the expression of genes related to intestinal barrier function via qPCR. As illustrated in Figure 5, group M demonstrated reduced gene expression levels of *ZO-1*, *Occludin*, *Claudin-1*, and *MUC-2* in comparison to group C (*p* < 0.01). The relative expression levels of *ZO-1*, *Occludin*, *Claudin-1*, and *MUC-2* genes in group CPE were considerably elevated (*p* < 0.01 or *p* < 0.05) compared to those in group M.

### 3.6. CPE Modulated the Overall Structure of Gut Microbiota in Cy-Treated Mice

Fecal samples were collected for subsequent 16S rDNA sequencing to examine gut microbiota. A total of 1,777,588 highly qualified reads were obtained, averaging 74,066 reads per sample. Operational Taxonomic Units (OTUs) were delineated using a 97% pairwise identity criteria.

As illustrated in Table 3, in comparison to group C, the Chao1, Shannon, and Simpson indices in group M exhibited an upward tendency; however, no statistically significant difference was observed (*p* > 0.05). The Chao1 index was markedly reduced in group CPE compared to group M (*p* < 0.05). Moreover, the Chao1, Shannon, and Simpson indices in the CPE group were more similar to those of the control group.

**Figure 6:**
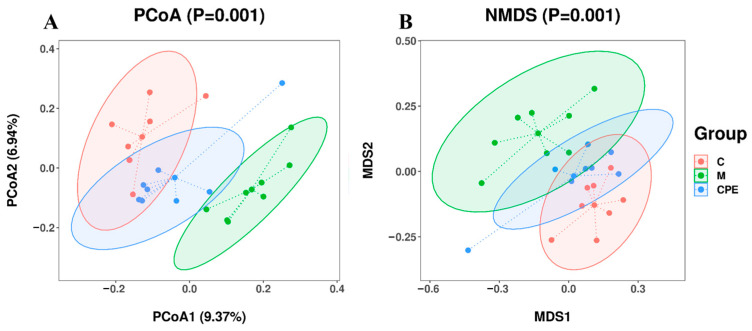
Beta diversity index. (**A**) Principal coordinates analysis (PCoA). (**B**) Nonmetric multidimensional scaling (NMDS). (*n* = 8).

Figure 6 illustrates a markedly significant separation trend across the samples within each category. Group M exhibited a significant divergence from group C, indicating a disparity in species composition between the two groups. Furthermore, group CPE demonstrated a closer connection to group C.

**Figure 7:**
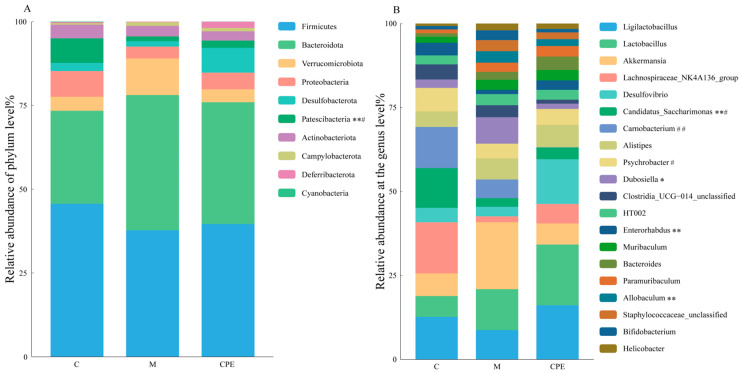
Detection results of microbial community composition in each group of experimental mice. (**A**) Relative abundance of microbes at the phylum level. (**B**) Relative abundance of microbes at the genus level. * indicates that group M exhibited statistical significance compared to group C. * indicates *p* < 0.05, ** indicates *p* < 0.01. # indicates that group CPE exhibited statistical significance compared to group M. # indicates *p* < 0.05, ## indicates *p* < 0.01 (*n* = 8).

As shown in Figure 7A, at the phylum level, *Firmicutes*, *Bacteroidota*, and *Verrucomicrobiota* were the main phyla. The relative abundances in each group were 45.61%, 27.76%, and 4.15% in group C; 37.73%, 40.30%, and 10.90% in group M; and 39.59%, 36.31%, and 3.87% in group CPE. In group M compared to group C, the relative abundance of *Firmicutes* decreased (*p* > 0.05) and *Patescibacteria* decreased significantly (*p* < 0.01), while *Bacteroidota* and *Verrucomicrobiota* increased (*p* > 0.05). In the CPE group, the relative abundance of *Firmicutes* increased (*p* > 0.05) compared to group M. Additionally, the relative abundance of *Patescibacteria* increased significantly (*p* < 0.05), while *Bacteroidota* decreased (*p* > 0.05) and *Verrucomicrobiota* also decreased (*p* < 0.05).

The distribution of gut microbiota at the genus level in each group is shown in Figure 7B. The gut microbiota of mice was made up primarily of *Ligilactobacillus*, *Lactobacillus*, *Akkermansia*, *Lachnospiraceae_NK4A136_group*, *Desulfovibrio*, *Carnobacterium*, *Candidatus_Saccharimonas*, *Alistipes*, *Psychrobacter*, and *Dubosiella*. Among all classified genera, the abundance of *Ligilactobacillus* was the highest (group C: 10.02%, group M: 4.80%, group CPE: 7.84%). Compared to group C, the abundance of *Candidatus_Saccharimonas* and *Enterorhabdus* was significantly reduced in group M (*p* < 0.01). Additionally, *Carnobacterium* and *Psychrobacter* showed a decreasing trend (0.05 < *p* < 0.1), while the abundances of *Dubosiella* and *Allobaculum* were significantly higher (*p* < 0.01 or *p* < 0.05). *Akkermansia* exhibited a trend of increase (*p* > 0.05). In the CPE group, there was a significant increase in the abundance of *Candidatus_Saccharimonas* and *Psychrobacter* compared to group M (*p* < 0.05). Conversely, *Carnobacterium* abundance was significantly decreased (*p* < 0.01), and *Allobaculum* abundance tended to decrease (0.05 < *p* < 0.1).

**Figure 8:**
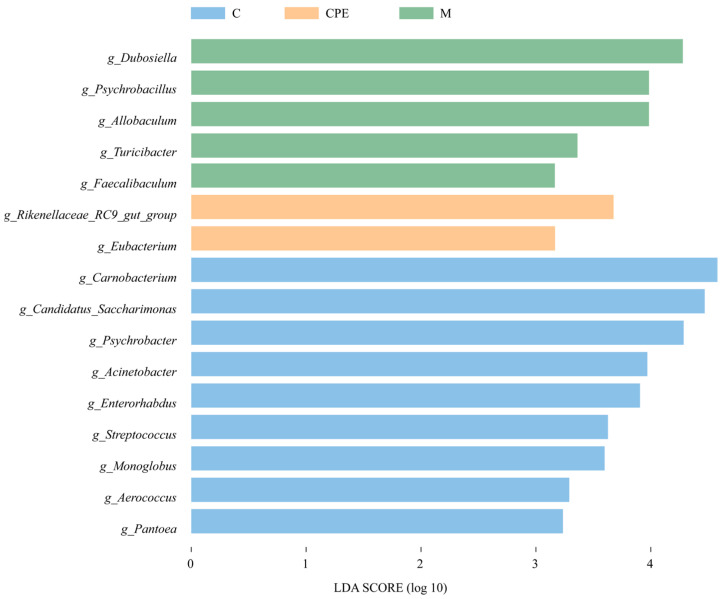
Linear discriminant analysis score (*n* = 8).

To identify the key phylotypes of gut bacteria across different groups, we analyzed species differences using LDA, combining LEfSe algorithm and *p*-values to assess the impact of species abundance on the variation among the groups. We screened for differential species with LDA score ≥ 3 and *p* < 0.05. As shown in Figure 8, group C exhibited a greater number of bacterial groups compared to group M, while group CPE had the fewest. The primary bacterial groups identified in group C included *Carnobacterium*, *Candidatus_Saccharimonas*, *Psychrobacter*, *Acinetobacter*, *Enterorhabdus*, *Pantoea*, *Streptococcus*, *Monoglobus*, and *Aerococcus*. The main bacterial groups identified in group M included *Dubosiella*, *Psychrobacillus*, *Allobaculum*, *Turicibacter* and *Faecalibaculum*. The main bacterial groups identified in group CPE included *Rikenellaceae_RC9_gut_group* and *Eubacterium*.

## 4. Discussion

### 4.1. Effect of CPE on Body Weight in Mice

Cy can cause immunosuppression and induce body weight loss in mice [21,22,23]. The results of this study showed a significant decrease in body weight in mice of group M after intraperitoneal injection of Cy, which aligned with the aforementioned research findings. Furthermore, the body weight of mice in the CPE-treated group was higher than that of mice in group M and was similar to the body weight of mice in the normal group, indicating that CPE mitigated Cy-induced body weight loss. This may be related to the fact that the main components of CPE are small-molecule active peptides, which can rapidly provide energy to the body by supplementing amino acids, regulating appetite, and improving intestinal villus morphology, thereby promoting growth [24]. For instance, shrimp peptide hydrolysates and bioactive liver peptides have been shown to improve food intake and increase body weight in immunosuppressed mice [25,26], which is consistent with the results of this study.

### 4.2. Effect of CPE on Immune Function in Mice

The thymus and spleen are essential immunological organs in the body [27]. A reduction in the weight of these organs is indicative of a compromised immune function [28,29,30]. In this study, the spleen and thymus index of mice in group M were significantly reduced, indicating that an immunosuppression model was successfully established. Conversely, the thymus and spleen index of mice in group CPE were significantly higher than those in group M, suggesting that CPE promotes the recovery of spleen and thymus functions, and enhances immune function. Cytokines serve as crucial mediators and regulators of the immune response, and their secretion levels can reflect the body’s immune function [19]. Additionally, studies have reported that IL-4, IL-10, and TNF-α are closely associated with the production of mucosal sIgA [31,32]. In this experiment, intraperitoneal injection of Cy significantly reduced the levels of TNF-α, IL-1β, IL-4, IL-10, sIgA, and β-DF in the intestinal mucosa compared to normal mice, indicating a disruption of intestinal immune homeostasis. Bioactive peptides have demonstrated immunomodulatory effects. For example, ovotransferrin has been reported to promote the secretion of various cytokines (IL-4, IL-10, TNF-α, and INF-γ) and sIgA and counteract Cy-induced intestinal injury [33]. In this experiment, administration of CPE in mice increased the levels of TNF-α, IL-1β, IL-4, IL-10, and sIgA, indicating that CPE promoted the recovery of cytokines in the intestinal mucosa and promoted humoral immunity, thereby ameliorating CY-induced intestinal injury in mice. This is consistent with findings that CPE enhances the immune organ index, indicating that it may regulate immune cell activation and thus promote cytokine release. Although the changes in β-DF levels in the CPE-treated group did not reach statistical significance, there was a trend toward increased secretion. β-DF is primarily produced by intestinal epithelial cells [34]. This phenomenon may be associated with our histological analysis results, which demonstrated that Cy injections in mice caused severe intestinal tissue damage, which was partially repaired by CPE administration.

### 4.3. Effect of CPE on the Organization of Intestinal Mucosa in Mice

The small intestine is the primary site for nutrient digestion and absorption, and the morphology of the intestinal mucosa is a crucial indicator of intestinal health [35]. This study demonstrates that Cy administration significantly impacts the structural integrity of the small intestine, characterized by villus breakage, villus atrophy, and crypt destruction, indicating compromised intestinal health. In contrast, mice treated with CPE exhibited significant improvements in small intestine morphology, characterized by increased villus length, decreased crypt depth, and an elevated V/C ratio. This aligns with the observed body weight gain following CPE treatment. Moreover, compound small peptides derived from traditional Chinese medicine have demonstrated the ability to enhance intestinal mucosal morphology and augment crypt secretion function in Cy-induced immunosuppressed mice, consequently restoring normal intestinal absorption function [17]. In conclusion, CPE repaired villus damage, thereby regulating intestinal absorption functions and supporting the growth and development of mice.

### 4.4. Effects of CPE on Intestinal Oxidation Indicators in Mice

Intraperitoneal injection of CY induced oxidative damage in the intestines [20]. SOD and MDA are typical oxidative stress markers, which can reflect the degree of oxidative injury in the intestinal tract [36]. In this experiment, intraperitoneal injection of CY caused an increase in MDA levels and a decrease in SOD activity in intestines, indicating that the intestinal tract was in a state of oxidative stress. According to reports, oyster peptides have been shown to significantly increase SOD mRNA expression levels and decrease MDA levels in the intestines of immunosuppressed mice [22]. The results of this study indicated that CPE administration increased SOD activity in the intestines of mice, suggesting that CPE could alleviate Cy-induced oxidative damage. Previous studies had found that CPE has good antioxidant function, which could regulate oxidative stress in the body by increasing serum CAT, GSH-Px, SOD, and GSH activities and decreasing MDA levels [15]. However, although the difference in MDA levels in the CPE group compared to the M group was not statistically significant, it showed a decreasing trend. This might have been because MDA, as a final product of lipid oxidation, is influenced by upstream oxidative pathways [37] and may have required more time to reflect the drug’s effect.

### 4.5. Effect of CPE on Intestinal the Barrier in Mice

DAO activity and D-LA levels can serve as indicators reflecting the integrity and degree of damage to the intestinal barrier [38,39]. Mucin 2 (MUC-2), produced by goblet cells, constitutes the chemical barrier adhering to epithelial surfaces, guarding against pathogenic intrusion [40]. Claudin-1, Occludin, and ZO-1 are the principal tight junction proteins that can maintain tight junction integrity and are crucial for maintaining intestinal barrier function [41]. In this study, compared to the normal group, intraperitoneal injection of Cy increased serum DAO activity and D-LA levels in mice, while the relative mRNA expression levels of *ZO-1*, *Occludin*, *Claudin-1*, and *MUC-2* in the intestine were reduced. This finding is consistent with the results reported by Cai et al. [20], indicating that Cy administration disrupts the intestinal barrier in mice. Bioactive peptides can act as trophic factors, providing energy to tight junction proteins by increasing protein synthesis [42]. Shrimp peptide hydrolysate restores mouse barrier integrity by restoring goblet cell populations and increasing tight junction protein production [25]. The results of this study showed that in the CPE treatment group, serum DAO activity and D-LA levels decreased, while the mRNA expression levels of *ZO-1*, *Occludin*, and *MUC-2* in the intestine increased. This suggested that CPE enhanced the integrity of tight junctions by regulating intestinal barrier gene expression, thereby effectively alleviating Cy-induced intestinal barrier dysfunction.

### 4.6. Effect of CPE on the Intestinal Microbiota in Mice

The intestinal tract is a complex microbial ecosystem, wherein microbes engage in diverse interactions with the host, significantly influencing the regulation of the host’s immune system via bacterial components and metabolites [43]. Therefore, we studied the effect of CPE on the intestinal microbes of Cy-treated mice using 16S rDNA sequencing technology. Our study showed that significant changes in microbial community beta diversity were observed after Cy administration, which was consistent with the existing research results [44]. CPE can mitigate these negative effects, illustrated by the fact that the structure of the CPE group was more similar to the control group. Furthermore, CPE treatment can restore microbial community diversity.

At the phylum level, *Firmicutes* and the *Bacteroidota* were the main dominant microbes in the mouse gut [3,45]. In this study, compared with normal group mice, the abundance of *Firmicutes* in group M was reduced, the abundance of *Bacteroidota* was increased, and the ratio of *Firmicutes*/*Bacteroidota* (*F*/*B*) was reduced. A decrease in the F/B ratio may lead to an imbalance between regulatory T cells and Th17 cells, resulting in dysfunction of the intestinal immune system [46]. Additionally, the relative abundance of *Patescibacteria* was reduced in group M compared to group C. This organism mainly exists in anaerobic environments, so it is hypothesized that the decrease in abundance was due to oxidative stress in the intestine of Cy-injected mice in the early stage, which destroyed the anaerobic environment required for the growth of *Patescibacteria*. Then, in the late stage, the infection is dominated by *Bacteroidota*, which inhibits its proliferation. In contrast, CPE can improve the intestinal barrier function, reduce the level of oxidative stress, and maintain the anaerobic environment of the intestinal tract, which is conducive to the recovery of *Patescibacteria* abundance.

At the genus level, group M had a high abundance of the genera *Dubosiella*, *Allobaculum*, and *Akkermansia*. Cy can cause a decrease in the abundance of *Akkermansia*, as reported by Yue [47] and Zhang [48]. While *Akkermansia* plays a role in regulating host immune responses [49], it is also a mucin-degrading bacterium, and excessive levels can compromise the mucus layer [50]. Thus, balancing its abundance is critical to maintaining intestinal health. Furthermore, studies have shown that *Allobaculum* facilitates the production of short-chain fatty acids (SCFA) in the intestinal tract, which can play an anti-inflammatory role [51]. *Dubosiella* improves oxidative stress and increases beneficial bacteria such as *Lactobacillus* and *Bifidobacterium* [52]. This may be due to the fact that Cy was injected during the last three days of the trial in this study, and the damage to the intestinal homeostasis was shallow. The presence of beneficial bacteria may indicate an attempt by the gut microbiome to re-establish balance, thereby enhancing the gut’s resilience to damage. CPE treatment was able to increase the relative abundance of *Candidatus_Saccharimonas* and *Psychrobacter*. *Candidatus_Saccharimona* maintains normal gut function and plays an important role in driving the mature host immune system [53]. The genus *Psychrobacter* is beneficial for improving nutrient utilization and innate immunity in the animal gut and plays a positive role in improving gut microbial diversity [54]. In summary, CPE promotes the remodeling of the intestinal microbiota in immunosuppressed mice induced by Cy, but the bacterial abundance of these mice showed some novel changes compared with normal mice, and these changes need to be further investigated.

## 5. Conclusions

**Figure 9:**
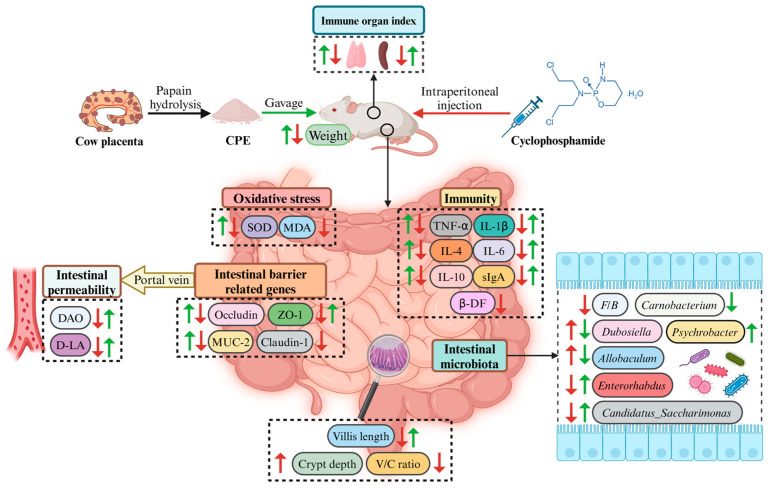
Cow placenta extract ameliorates cyclophosphamide-induced intestinal damage by enhancing the intestinal barrier and immune function and restoring intestinal microbes. Note: The green arrows indicate the effect of CPE treatment, and the red arrows indicate the effect of Cy administration on mice. ↑: upregulation; ↓: downregulation. The figure was created using BioRender.

This study reports the beneficial effects of CPE in preventing intestinal damage induced by Cy in immunosuppressed mice, confirming that CPE mitigates the intestinal damage caused by Cy, raises the immune organ index, and increases the levels of intestinal immune-related cytokines, secretory sIgA, and β-DF, consequently enhancing the immune function of the intestinal mucosa. Moreover, CPE counteracts the intestinal barrier breakdown and oxidative damage caused by Cy, hence decreasing intestinal permeability. In addition, CPE mitigates the dysbiosis of gut microbiota caused by Cy and restores the balance of microbial ecology (Figure 9). This provides a new approach for its clinical use in the treatment of immunosuppression-induced intestinal damage.

## Figures and Tables

**Table 1:** Sequences of the primers used for RT-PCR.

Gene	Forward Primers (5′-3′)	Reverse Primers (5′-3′)	Size (bp)
*GAPDH*	CCTCGTCCCGTAGACAAAATG	TGAGGTCAATGAAGGGGTCGT	133
*Mucin-2*	TCCTGACCAAGAGCGAACAC	ACAGCACGACAGTCTTCAGG	102
*ZO-1*	GCCGCTAAGAGCACAGCAA	GCCCTCCTTTTAACACATCAGA	103
*Occludin*	TCTGCTTCATCGCTTCCTTAGT	AGCCGTACATAGATCCAGAAGC	189
*Claudin-1*	TGGTAATTGGCATCCTGCTG	CAGCCATCCACATCTTCTGC	122

**Table 2:** Changes in body weight of mice during the experiment.

Time		Group	
	C	M	CPE
Day 0	43.81 ± 0.36 _a_	43.66 ± 0.32 _a_	43.79 ± 0.34 _a_
Day 7	45.41 ± 0.32 _b_	45.73 ± 0.28 _b_	45.58 ± 0.44 _b_
Day 14	45.95 ± 0.37 _b_	46.13 ± 0.44 _bc_	46.56 ± 0.43 _bc_
Day 21	47.54 ± 0.36 _c_	47.64 ± 0.39 _c_	47.96 ± 0.42 _c_
Day 28	48.34 ± 0.64 _c_	46.03 ± 0.40 _b_^a^	47.27 ± 0.39 _c_^b^

Note: C = control group; M = cyclophosphamide model group; CPE = cow placental extract treatment group. For the same indicator, the superscript character represents intergroup differences, the subscript character represents intragroup differences, the same character indicates insignificant differences *p* > 0.05, and different characters indicate significant differences *p* < 0.05 (*n* = 12).

**Table 3:** Alpha diversity index.

Index	Group		
	C	M	CPE
Chao1	592.48 ± 51.75 ^ab^	703.68 ± 48.21 ^b^	582.40 ± 90.06 ^a^
Shannon	5.71 ± 0.38	6.13 ± 0.27	5.74 ± 0.32
Simpson	0.90 ± 0.03	0.93 ± 0.02	0.92 ± 0.02

Note: C = control group; M = cyclophosphamide model group; CPE = cow placental extract treatment group. For the same indicator, the superscript character represents intergroup differences, the same character indicates insignificant differences *p* > 0.05, and different characters indicate significant differences *p* < 0.05 (*n* = 8).

## Data Availability

The data presented in this study are available in the article/Supplementary Materials; further inquiries can be directed to the corresponding authors. All sequencing data are available through the NCBI Sequence Read Archive under the accession number PRJNA1166846.

## Supplementary Materials

The following supporting information can be downloaded at: https://www.mdpi.com/article/10.3390/vetsci11100505/s1, Figure S1: Results of body weight and immune organ index in preliminary experimental mice; Figure S2: Results of serum DAO in preliminary experimental mice; Figure S3: Results of intestinal sIgA in preliminary experimental mice.
