# Supplementary material for: Cow Placenta Extract Ameliorates Cyclophosphamide-Induced Intestinal Damage by Enhancing the Intestinal Barrier, Improving Immune Function, and Restoring Intestinal Microbiota
Source: Vet Sci. 2024 Oct 14;11(10):505. doi: 10.3390/vetsci11100505 (PMC11512425; doi:10.3390/vetsci11100505)
Supplement: Supplementary file 1 [file vetsci-11-00505-s001.zip › vetsci-3217195-supplementary.pdf]

# **Supplementary Materials:** Cow Placenta Extract Ameliorates Cyclophosphamide-Induced Intestinal Damage by Enhancing the Intestinal Barrier, Improving Immune Function, and Restoring Intestinal Microbiota

## 1 Supplementary Data

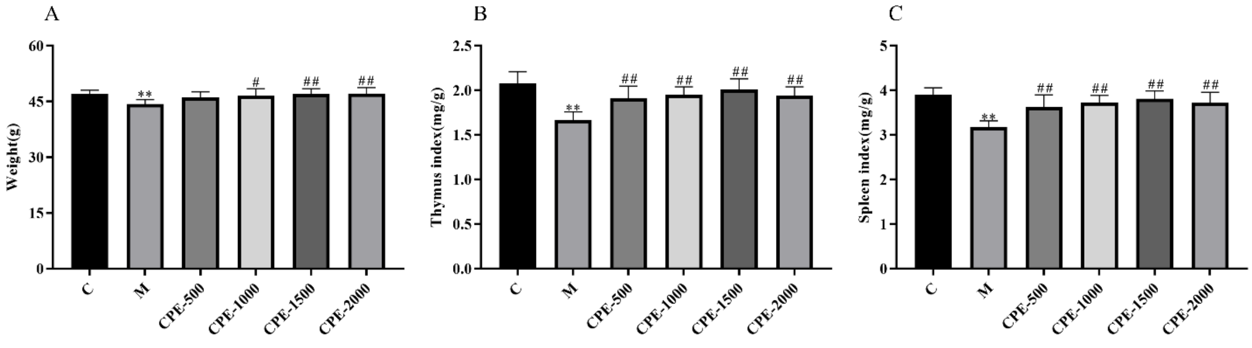


**Figure S1.** Results of body weight and immune organ index in preliminary experimental mice. (A) Mouse weight on the 28th day. (B) Thymic index in each group. (C) Spleen index in each group. "*" indicates whether there is statistical significance compared to group C, "*" indicates P<0.05, "**" indicates P<0.01; "#" indicates whether there is statistical significance compared to group M, "#" indicates P<0.05, "##" indicates P<0.01. The same below. (n=9).

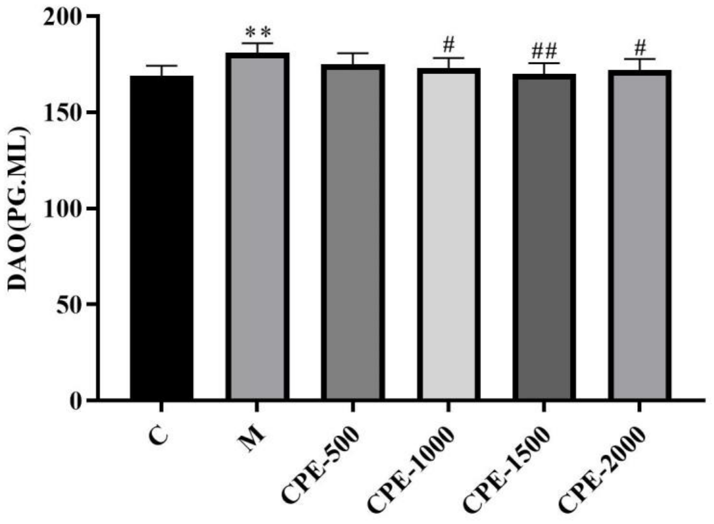


**Figure S2.** Results of serum DAO in preliminary experimental mice. (n=9).

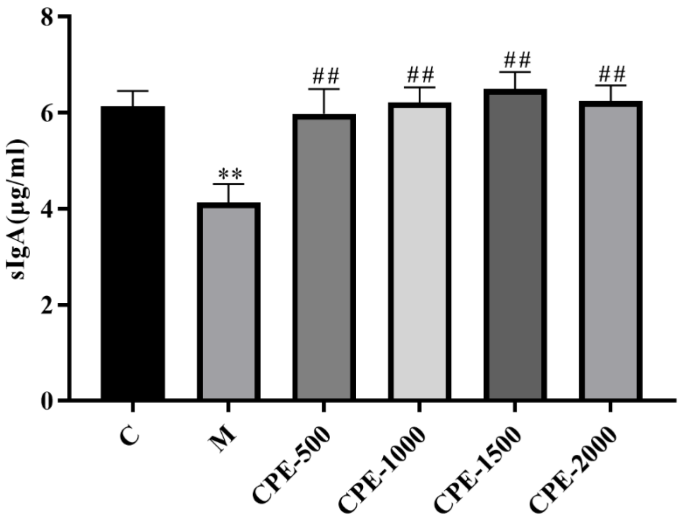


**Figure S3.** Results of intestinal sIgA in preliminary experimental mice. (n=9).
